# Supplementary material for: Local features drive identity responses in macaque anterior face patches
Source: Nat Commun. 2022 Sep 23;13:5592. doi: 10.1038/s41467-022-33240-w (PMC9508131; doi:10.1038/s41467-022-33240-w)
Supplement: Supplementary file 1 — Supplementary Information [file 41467_2022_33240_MOESM1_ESM.pdf]

Extended Data

Supplementary Table 1. Number of parts-selective neurons in each face patch

	MA (n=44)	WA (n=36)	MO (n=36)	SP1 (n=37)	SP2 (n=55)
Eyes-selective	22 (50%)	24 (66%)	0 (0%)	1 (3%)	1 (1%)
Mouth-selective	0 (0%)	4 (11%)	19 (52%)	0 (0%)	0 (0%)
Outer face-selective	9 (20%)	3 (8%)	8 (22%)	19 (51%)	25 (45%)

(AM blue, AF red)

Supplementary Table 2. Image Sources and Licenses

Stimulus	Figure*	License	Source
Monkey 1	1a, 1b^{1}, 1d-e, 2a-b, 4a, 4d, 5a-b, S1c-d, S4c, S5a	CC-BY 4.0	https://figshare.com/articles/dataset/Macaque_Faces/9862586/1 Umber-9211-160506.jpg
Monkey 2	1b^{1}, 2a, S1a-b	CC-BY 4.0	https://figshare.com/articles/dataset/Macaque_Faces/9862586/1 Beowulf-9372-180329.jpg
Monkey 3	1b^{3}, 2a, S1a-d	CC-BY 4.0	https://figshare.com/articles/dataset/Macaque_Faces/9862586/1 Whistler-3609-160118.jpg
Monkey 4	1b^{4}, 2a	CC-BY 4.0	https://figshare.com/articles/dataset/Macaque_Faces/9862586/1 Brandy-5322-171018.jpg / Brandy-5314-171018.jpg
Monkey 5	1b^{5}, 2a, 5a-b, S1c-d	CC-BY 4.0	https://figshare.com/articles/dataset/Macaque_Faces/9862586/1 Jeremy-6920-140709.jpg
Monkey 6	1b^{6}	CC-BY 4.0	https://figshare.com/articles/dataset/Macaque_Faces/9862586/1 Zeb-1457-160804.jpg
Monkey 7	1b^{7}	CC-BY 4.0	https://figshare.com/articles/dataset/Macaque_Faces/9862586/1 Ash-2440-171222.jpg
Monkey 8	1b^{8}	CC-BY 4.0	https://figshare.com/articles/dataset/Macaque_Faces/9862586/1 Cato-0395-171222.jpg
Monkey 9	1b^{9}, S1a-b	CC-BY 4.0	https://figshare.com/articles/dataset/Macaque_Faces/9862586/1 Spangle-2792-170714.jpg
Monkey 10	1b^{10}, 1d, 5a-b, S4c	CC-BY 4.0	https://figshare.com/articles/dataset/Macaque_Faces/9862586/1 Versa-9926-170616.jpg
Scene 1 : ocean	1c^{i}, 1d, 1e, 2b, 4a, 4d,	n/a	Taken by authors JJH
Scene 2 : lab	1c^{ii}, 2a, 5b, S1c-d	n/a	Taken by author KWK
Scene 3 : nih	1c^{iii}, 2a, 5b	n/a	Taken by author KWK
Scene 4 : fall	1c^{iv}, S1a-b	n/a	Taken by author KWK
Scene 5 : snow	1c^{v}, 2a, 5b, S1a-d	n/a	Taken by author ENW
Scene 6 : farm	1c^{vi}, S1a-b	n/a	Taken by author ENW
Scene 7 : lake	1c^{vii}, 2a, 5b	n/a	Taken by author ENW
Scene 8 : nyc	1c^{viii}, 2a, 5a-b, S1c-d	n/a	Taken by author ENW
Scene 9: treewalk	1c^{ix}, 2a,	n/a	Taken by author ENW
Scene 10: overlook	1c^{x}, 1d, 2a,	n/a	Taken by author JJH

* Figure(s) that the stimulus image is found in. Superscripts for Fig. 1b and 1c signify the left-to-right position within the set of monkey or scene images present (e.g. Fig. 1b^{1} means the leftmost monkey in Fig. 1b)

Supp. Figure 1

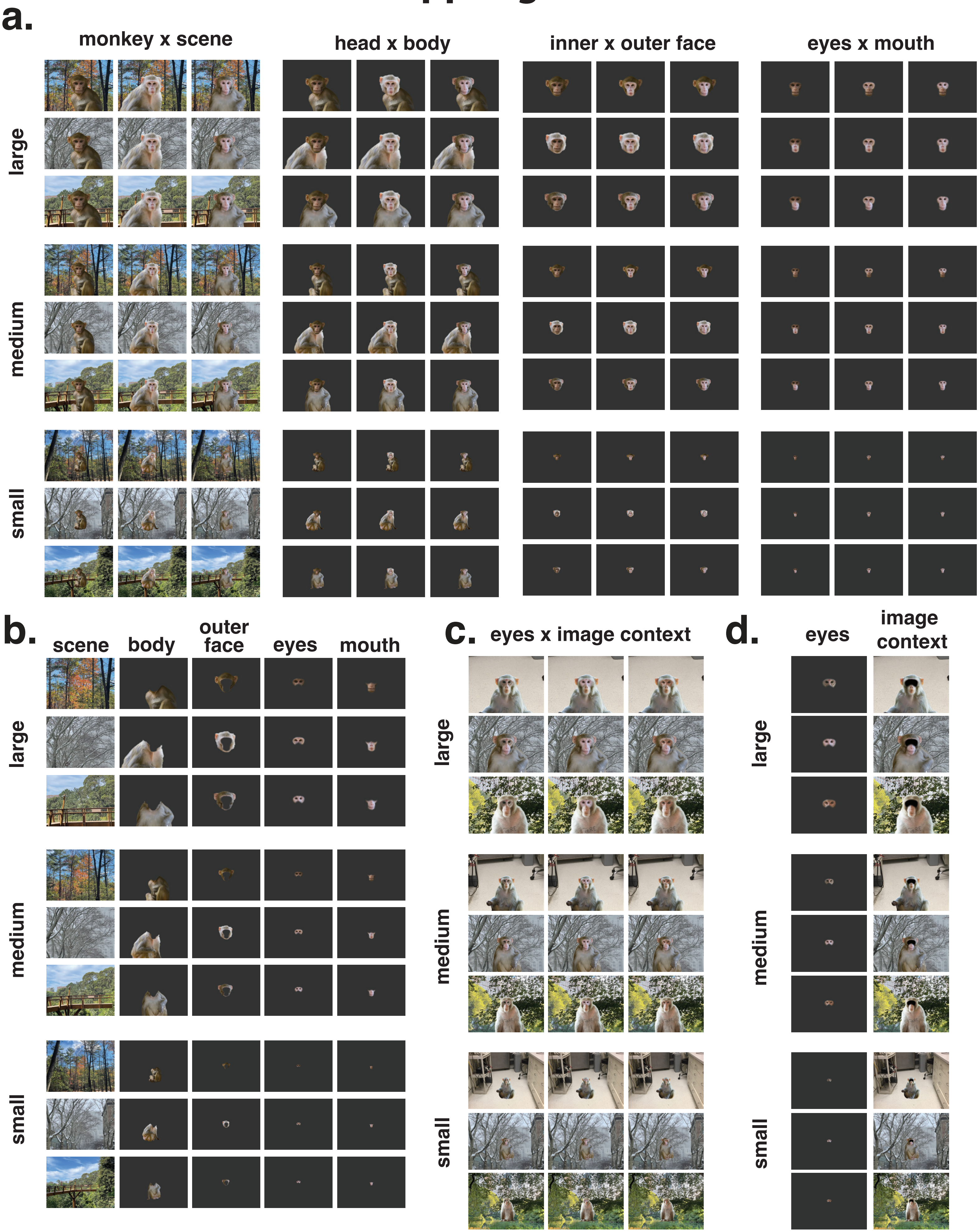

Figure S1: Example Face-swapping Stimuli

(a) Example stimuli, showing systematic recombination of image parts across the four swapping categories (columns) for monkeys 2, 3, and 9 and scenes iv, v, and vi, across the three different sizes (rows). **(b)** Parts alone stimuli (columns) for monkeys and scenes in (a) across three sizes (rows). **(c)** Eyes × image context experimental stimuli: systematic recombination of eyes and image context stimuli for monkey/scene pairs 1/ii, 3/v, and 5/viii. **(d)** Parts alone for eyes × image context stimuli in (c). Macaque images in (a-d) were obtained from https://figshare.com/articles/dataset/Macaque_Faces/9862586/1 under a CC-BY 4.0 license. Stimuli shown here are representative of those originally presented, not the originals.

Supp. Figure 2

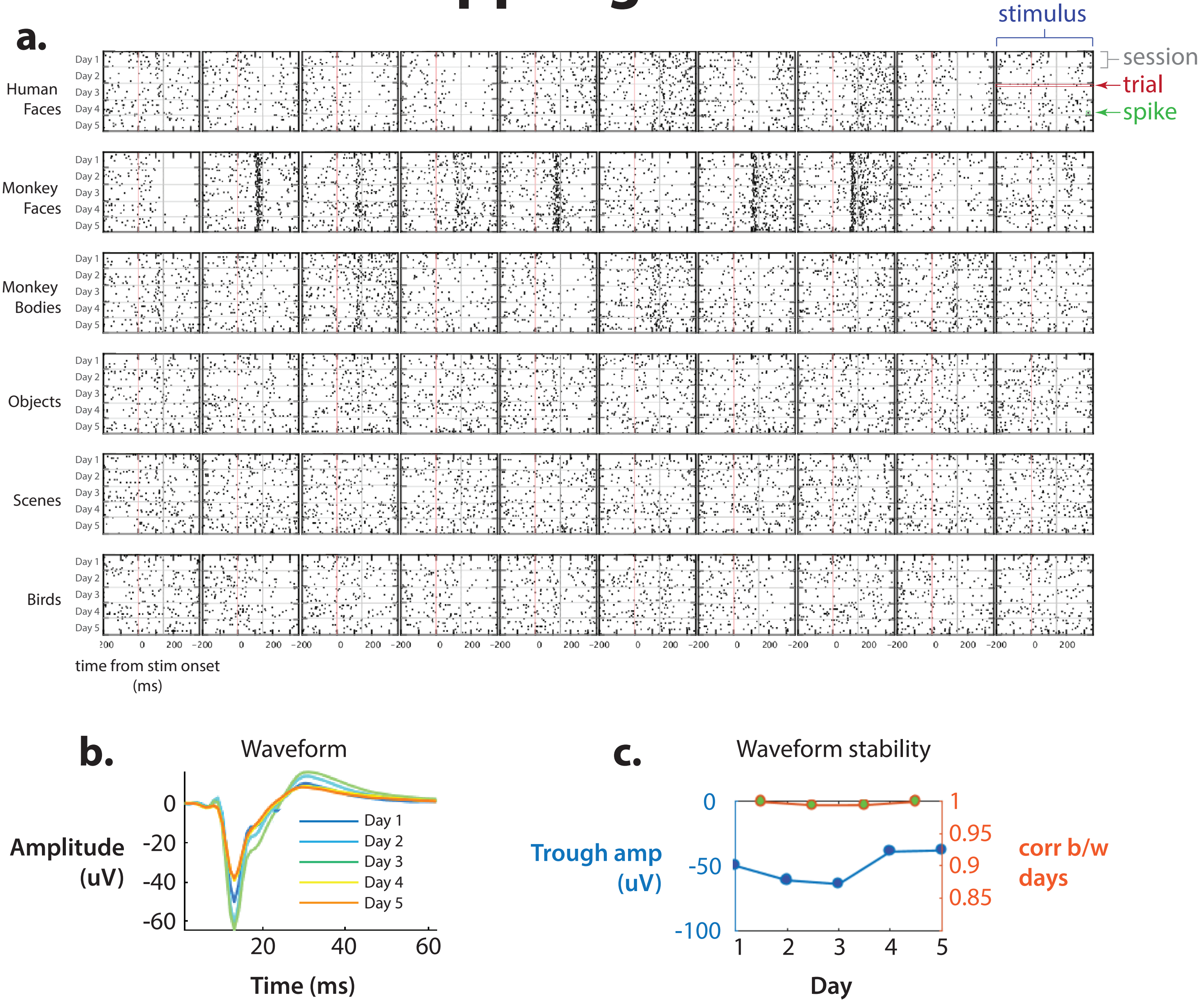

Figure S2: Tracking neurons across days

(a) Raster plots for one cell, MA 006_2, across five recording sessions, in response to 60 'Fingerprinting' stimuli. Stimuli are each displayed at least 10x to monkeys after each day of recording and are used to assess response consistency of each neuron across days-weeks. Firing rate and response selectivity remains consistent across days. **(b)** Action potential waveforms of the neuron in (b) across all recording sessions; amplitude and shape remain constant. **(c)** Trough amplitude is consistent throughout recording, and correlation between days is close to 1.

Supp. Figure 3

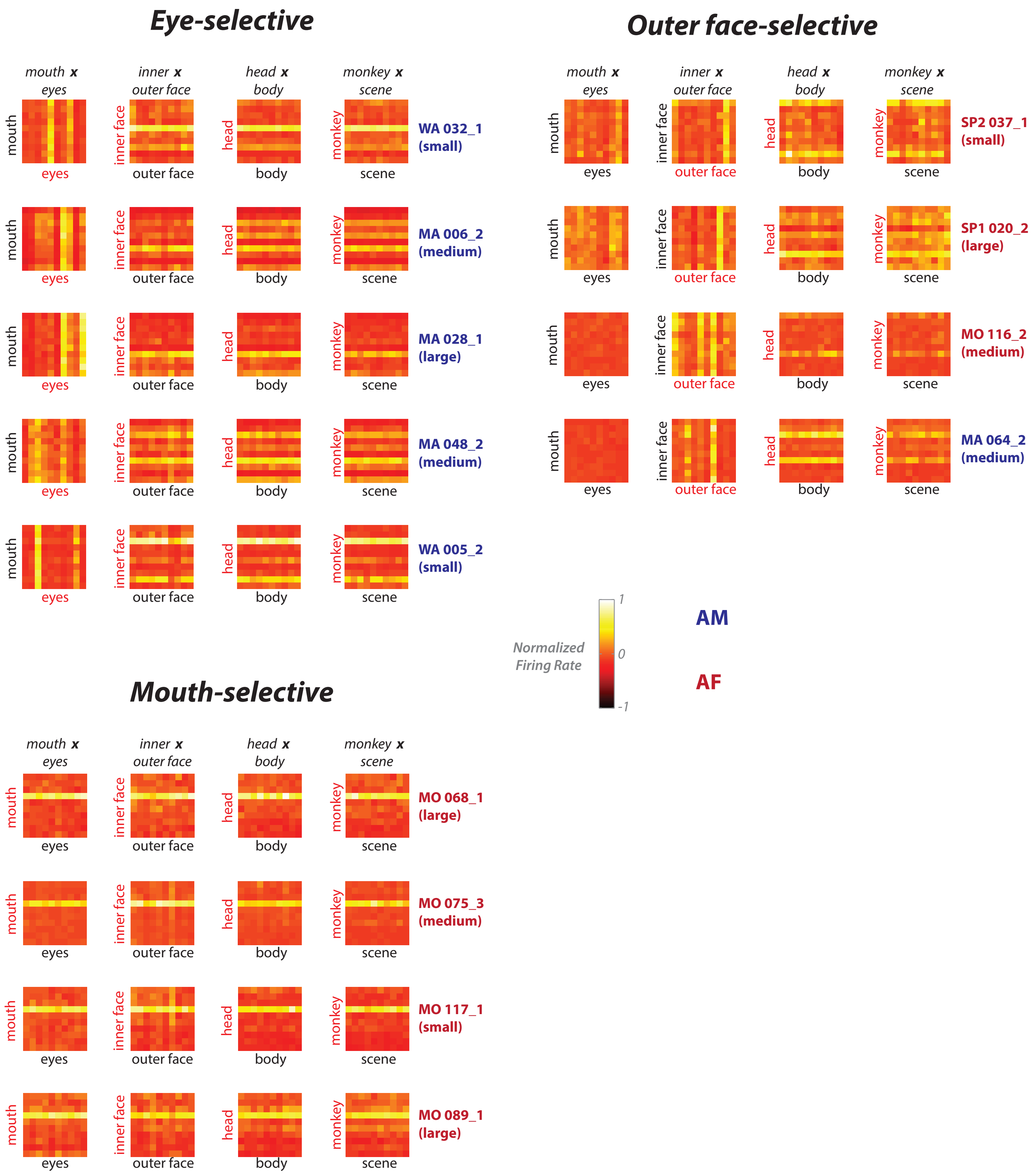

Figure S3: Examples of Part-selective Neurons

Examples of eyes-selective, mouth-selective, and outer-face-selective cells. Neurons from AM are marked in blue, AF in red. Responses to *mouth* × *eyes, inner* × *outer face, head* × *body,* and *monkey* × *scene* stimuli are shown for each cell, and the preferences for the local part of interest carried through each larger image context and drive the response preferences at the whole image level.

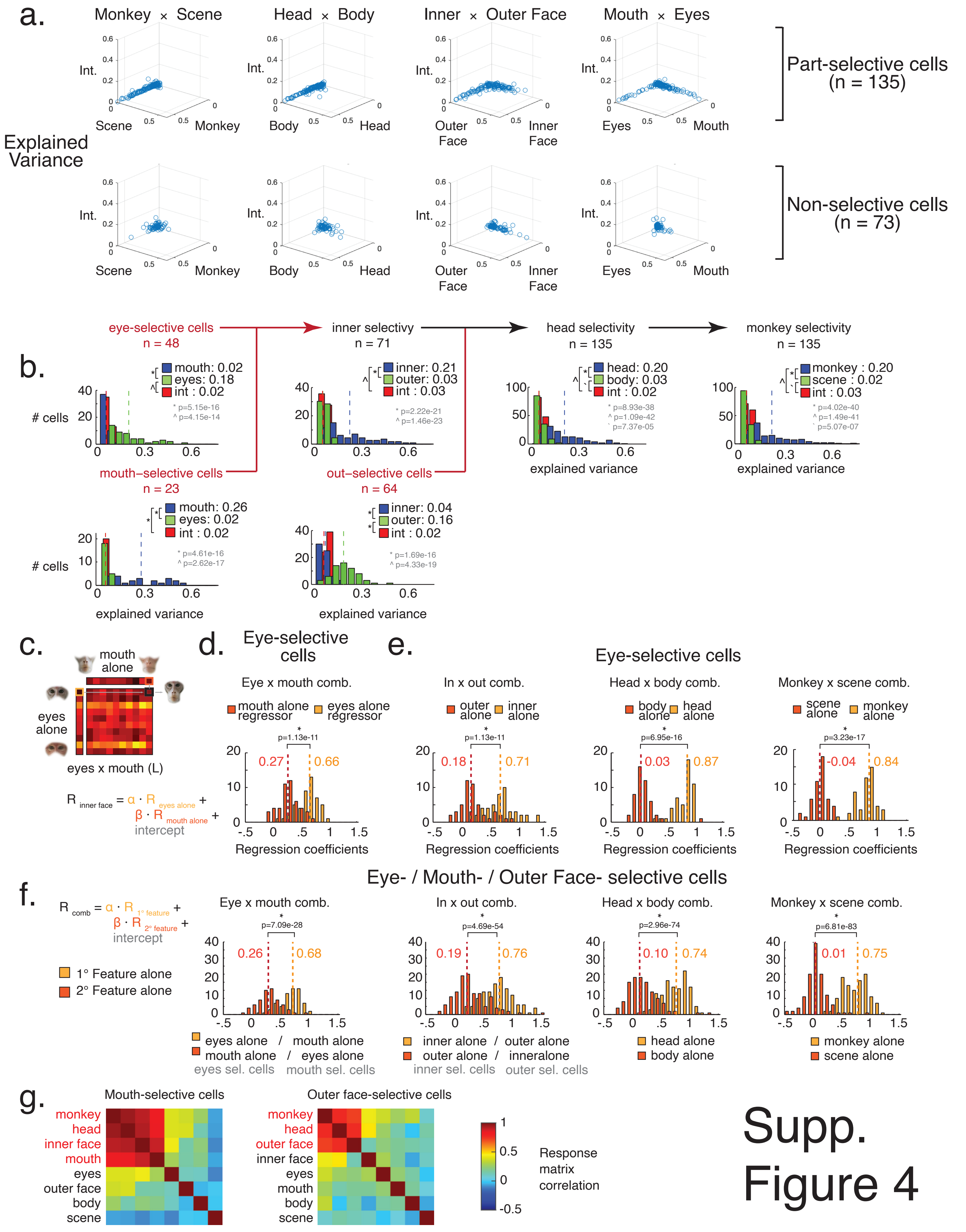

Supp. Figure 4

Figure S4: Tuning profiles of Part-selective Neurons

(a) Explained variance (2-way ANOVA) for each factor (e.g. eyes, mouth, interaction for *eye* × *mouth* stimuli, far right column) across all swapping categories, for all parts-selective (n=135, top row) and all non-parts-selective (n=73, bottom row) neurons. Non-parts-selective cells also do not appear to show any strong conjunctive or holistic tuning. **(b)** Histogram of explained variance for eyes-selective, mouth-selective, and outer-face-selective neurons. Median of each distribution in black; p-values (2-sided Wilcoxon Rank Sum test) in grey. **(c)** Heatmap of responses to eyes alone, mouth alone, and *eyes* × *mouth* stimuli for one eyes-selective cell. Below, model for regression of eyes- and mouth-alone responses against combined *eyes* × *mouth* responses. Macaque images were obtained from https://figshare.com/articles/dataset/Macaque_Faces/9862586/1 under a CC-BY 4.0 license. **(d)** Distribution of regression coefficients for mouth-alone and eyes-alone responses regressed against *eyes* × *mouth* responses, as in (d), for eyes-selective cells. Responses to eyes-alone stimuli better predict the combined *eyes* × *mouth* response than do the mouth-alone responses (2-sided Wilcoxon Rank Sum, p values black text). **(e)** Regression analysis from (d) repeated for each subsequent swapping category for eyes- selective cells; parts alone responses that contain the primary feature (inner face, head, monkey) better predict the combined responses than do the secondary feature responses (outer face, body, scene) (2-sided Wilcoxon Rank Sum, p values black text). **(f)** Regression analysis in (d-e) for eyes-, mouth-, and outer-face-selective cells, combined. The primary and secondary feature for the parts-alone responses differ depending on the type of selectivity; for example, for *inner* × *outer face* stimuli, the primary feature is the inner face for eyes- and mouth-selective cells, and outer face for outer- face selective cells. Stimulus types containing the local part of interest (eyes, mouth, outer face) continue to better predict combined responses than do the other stimuli (2-sided Wilcoxon Rank Sum, p values black text). **(g)** Correlation matrices (as in Fig. 4c) for mouth- and outer-face-selective neurons. Correlation highest between matrices containing the preferred local part.

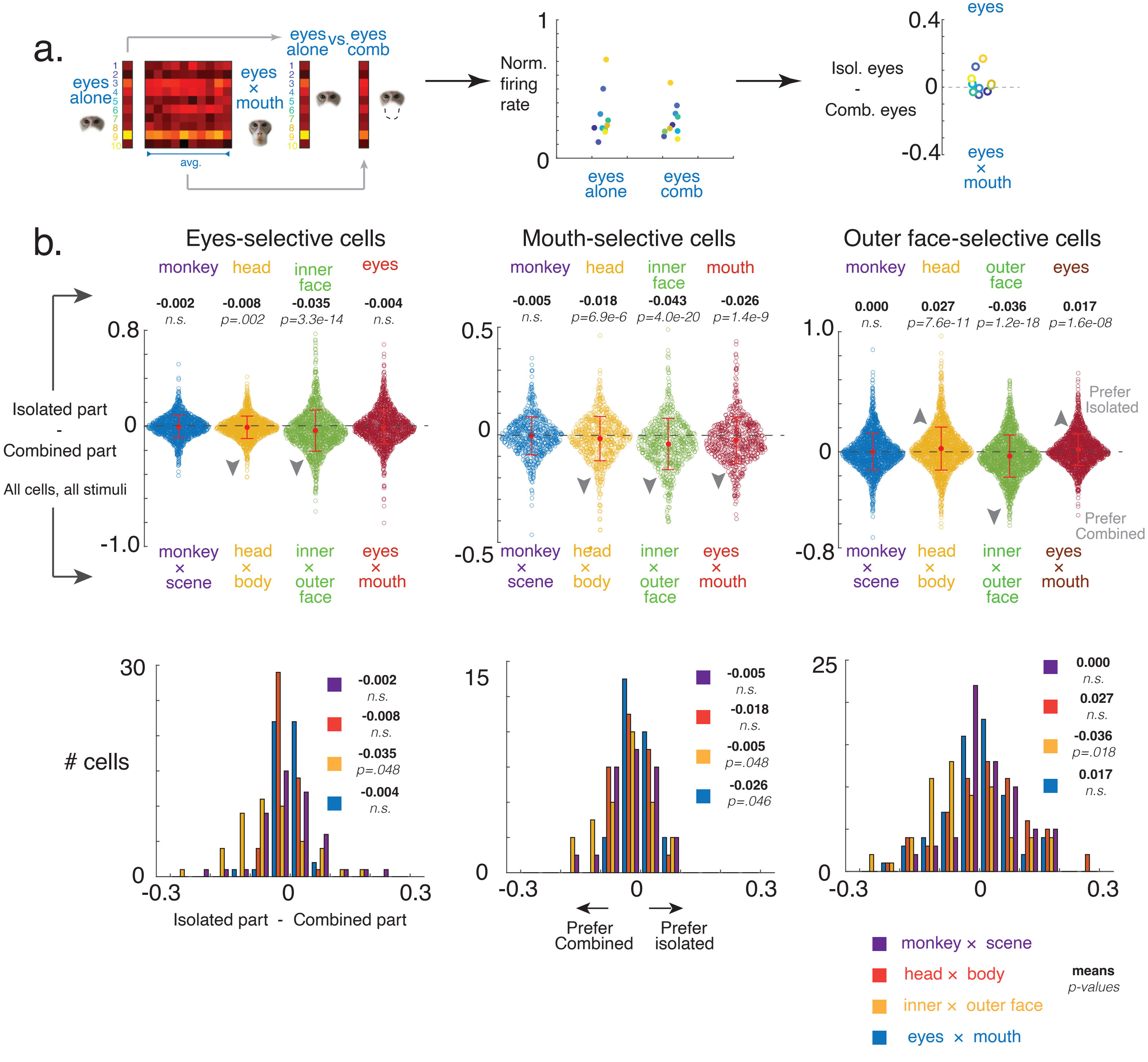

Supp. Figure 5

Figure S5: Isolated vs. Combined Features

(a) Left: example heatmap for one eyes-selective cell, of eyes alone responses compared to the average response for eyes across eyes × mouth stimuli ("eyes comb"). Middle: average normalized firing rate for eyes alone and in combination for each identity 1-10. Right: difference between eyes alone and eyes combined response for each identity 1-10. Macaque images were obtained from https://figshare.com/articles/dataset/Macaque_Faces/9862586/1 under a CC-BY 4.0 license. **(b)** Top row, swarm plots: Difference between part alone (colored text, above plot) and part combined (colored text, below plot) for all stimuli (as in (a)), across swapping categories and selectivity types (n = 48 eyes-selective cells, 23 mouth-selective cells, 64 outer-face-selective cells; x 30 stimuli each). Mean of each distribution in black bolded text, above plots. Red error bar is mean ± standard deviation. Some distributions significantly shifted from 0 (two-sided t-test, p values italicized text over plots). Significant preference for isolated (>0) or combined (<0) stimuli marked with grey arrow. Bottom row, histograms: For each cell, mean of differences between parts alone and parts combined for all stimuli, across swapping groups and selectivity types. Both plot types show general central tendency, indicating similar representation of parts alone and in combination, with each selectivity type containing some cells with preference for parts in isolation, and others with preference for parts in combination.

a. Eyes × image context, all cells

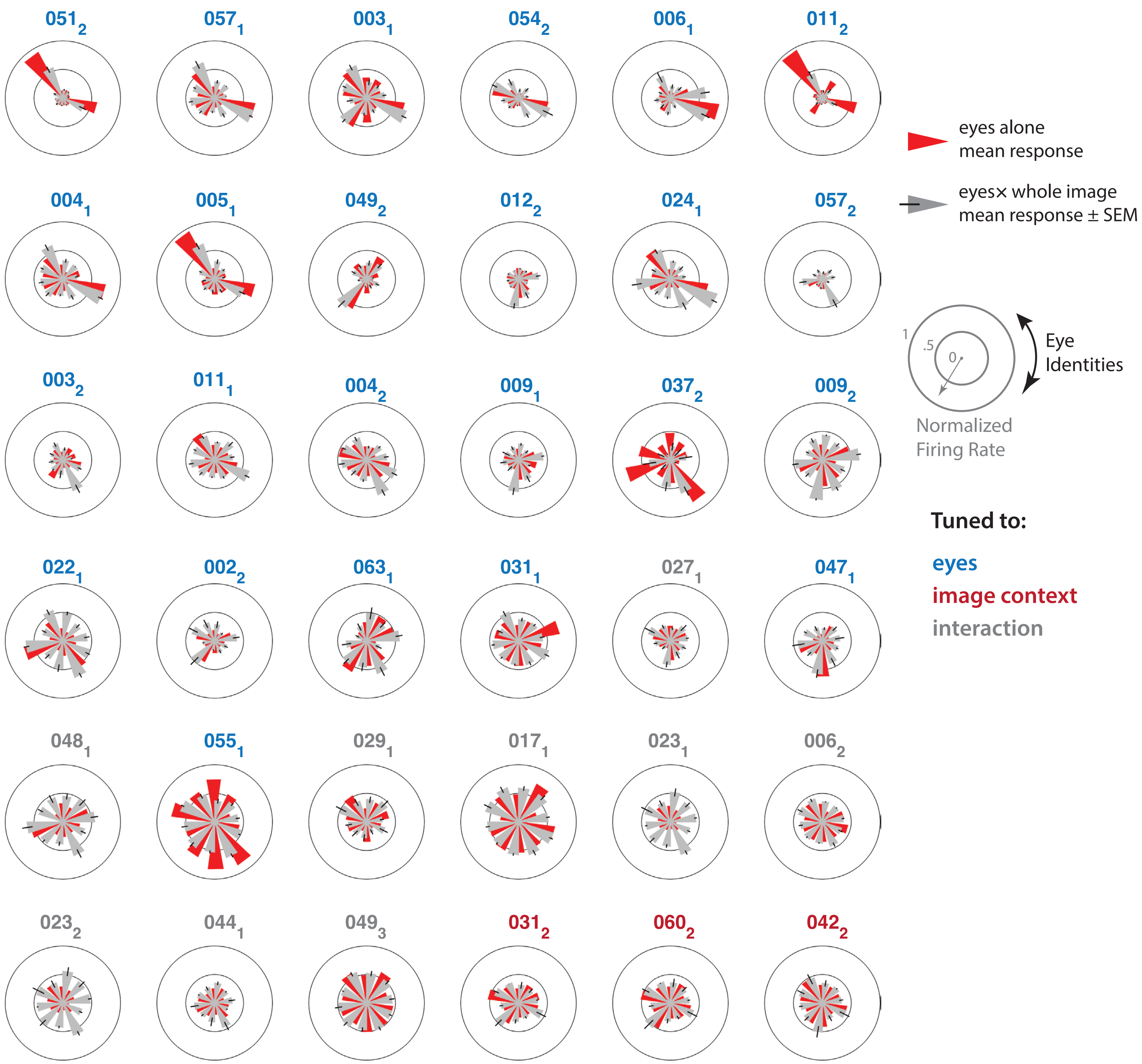

b.

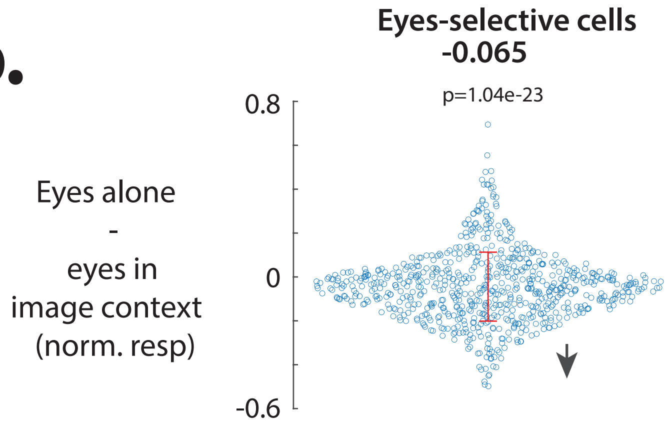

Supp. Figure 6

Figure S6: Radial plots for *Eyes × Whole Image* stimuli for all neurons

(a) Radial plots of eyes alone (mean, pink bar) and *eyes × image context* (mean, grey bar; SEM, black line) normalized responses for each identity, for all neurons at this AM site (monkey WA), including neurons tuned for eyes (blue labels), image context (red), and interaction (grey), sorted by preference (2-way ANOVA) for eyes. **(b)** Difference between eyes alone and eyes in combination (across image contexts) for all stimuli, all cells (n=36 cells x 30 stimuli each). Red error bar is mean ± standard deviation. Slight preference (two-sided t-test, p value over plot) for combined stimuli.
