# Supplementary material for: Local features drive identity responses in macaque anterior face patches
Source: Nat Commun. 2022 Sep 23;13:5592. doi: 10.1038/s41467-022-33240-w (PMC9508131; doi:10.1038/s41467-022-33240-w)
Supplement: Supplementary file 3 — Reporting Summary [file 41467_2022_33240_MOESM3_ESM.pdf]

nature portfolio

Corresponding author(s): Dr. David Leopold (leopoldd@mail.nih.gov)
Dr. Kenji Koyano (kenji.koyano@nih.gov)

Last updated by author(s): 05/14/2022

Reporting Summary

Nature Portfolio wishes to improve the reproducibility of the work that we publish. This form provides structure for consistency and transparency in reporting. For further information on Nature Portfolio policies, see our Editorial Policies and the Editorial Policy Checklist.

Statistics

For all statistical analyses, confirm that the following items are present in the figure legend, table legend, main text, or Methods section.

n/a	Confirmed	
☐	☒	The exact sample size (n) for each experimental group/condition, given as a discrete number and unit of measurement
☐	☒	A statement on whether measurements were taken from distinct samples or whether the same sample was measured repeatedly
☐	☒	The statistical test(s) used AND whether they are one- or two-sided<br>*Only common tests should be described solely by name; describe more complex techniques in the Methods section.*
☐	☒	A description of all covariates tested
☐	☒	A description of any assumptions or corrections, such as tests of normality and adjustment for multiple comparisons
☐	☒	A full description of the statistical parameters including central tendency (e.g. means) or other basic estimates (e.g. regression coefficient) AND variation (e.g. standard deviation) or associated estimates of uncertainty (e.g. confidence intervals)
☐	☒	For null hypothesis testing, the test statistic (e.g. F, t, r) with confidence intervals, effect sizes, degrees of freedom and P value noted<br>*Give P values as exact values whenever suitable.*
☒	☐	For Bayesian analysis, information on the choice of priors and Markov chain Monte Carlo settings
☒	☐	For hierarchical and complex designs, identification of the appropriate level for tests and full reporting of outcomes
☒	☐	Estimates of effect sizes (e.g. Cohen's d, Pearson's r), indicating how they were calculated

Our web collection on statistics for biologists contains articles on many of the points above.

Software and code

Policy information about availability of computer code

Data collection	Eye position was monitored with Eyelink (Ver 2.31). Electrophysiological data was collected and stored, along with data of event codes, eye positions, and a photodiode signal, using hardware and the following software from Tucker-Davis Technologies (TDT): OpenEX (Ver 2.31.0) or Synapse (Ver 92). Stimuli were created using Adobe Photoshop and Matlab (Ver 2014b-2021b) and presented with NIMH MonkeyLogic (Ver 2.2.20, on Matlab 2018a).
Data analysis	fMRI data was analyzed using AFNI (Cox 2012) and Matlab (Ver 2014b-2021b, MathWorks) software.<br>Spike-sorting of electrophysiological data was performed offline using WaveClus software (Quiroga et al. 2004). Subsequent analysis of neural activity was performed in Matlab using custom scripts, which are available in the figshare repository 10.6084/m9.figshare.19947182.

For manuscripts utilizing custom algorithms or software that are central to the research but not yet described in published literature, software must be made available to editors and reviewers. We strongly encourage code deposition in a community repository (e.g. GitHub). See the Nature Portfolio guidelines for submitting code & software for further information.

Data

Policy information about availability of data

All manuscripts must include a data availability statement. This statement should provide the following information, where applicable:

- Accession codes, unique identifiers, or web links for publicly available datasets
- A description of any restrictions on data availability
- For clinical datasets or third party data, please ensure that the statement adheres to our policy

The data generated in the current study are available in the figshare repository 10.6084/m9.figshare.19947188.

Field-specific reporting

Please select the one below that is the best fit for your research. If you are not sure, read the appropriate sections before making your selection.

- [x] Life sciences
- [ ] Behavioural & social sciences
- [ ] Ecological, evolutionary & environmental sciences

For a reference copy of the document with all sections, see nature.com/documents/nr-reporting-summary-flat.pdf

Life sciences study design

All studies must disclose on these points even when the disclosure is negative.

Sample size

Subjects: 4 individual macaque monkeys.
Two monkeys had electrodes implanted in face patch AM (monkeys WA and MA). Two monkeys had electrodes implanted in face patch AF (monkeys MO and SP (in this monkey, we recorded 2 distinct sites in the same face patch)).
N=2 monkeys per face patch was chosen to balance repeatability of data at each face patch with the associated costs, and is commonly used for nonhuman primate electrophysiology research.

Neurons: Of 403 single units (determined by cross-correlation spike timing analysis), 208 units were identified as face- and stimulus- selective and used in the final dataset.
80 from face patch AM: 44 from monkey MA, 36 from monkey WA.
128 from face patch AF: 36 from monkey MO, 37 from monkey SP site 1, 55 from monkey SP site 2.
These numbers of neurons in each face patch were not preselected, but determined by the population available across recordings and the proportion that ended up being face-selective. The numbers achieved are in line with other nonhuman primate electrophysiological studies.

To determine face- and stimulus-selectivity, we selected cells that met 2 criteria:
1) Face-selective: 1-way ANOVA for a significantly different response (baseline-subtracted) for the heads and monkeys in our stimulus set, relative to the bodies and scenes in isolation from our image set ($p<0.05$).
2) Responsive to some portion of our stimulus set: 1-way ANOVA, significant effect by stimulus on the baseline-subtracted neural response in at least one of 12 swapping categories (Bonferroni corrected, $p<0.0041$)

Data exclusions

Of the 403 unique single units collected, 195 cells were excluded for not meeting face- or stimulus-selectivity criteria.

Replication

For each face patch considered, single unit responses were collected from 2 different individual monkeys. The main effect described in the paper, in which many single neurons in these anterior patches are tuned largely to individual local parts, was observed across all 5 recording sites from the 4 monkeys. The results between the two AM monkeys were highly similar (with most neurons tuned to eyes) while the AF sites appeared more heterogeneous between monkeys with mouths or outer faces more strongly represented depending on the animal. However, in this paper we do not make claims about specific parts that AM and AF are broadly tuned to.

Randomization

The only specific consideration for which monkey was implanted in which face patch was implantation accessibility in each patch, and the needs for the lab of specific face patches to be recorded. So the selection of a monkey for a patch implantation was not purely random, but was not made with any expectation that a specific patch from a specific monkey would show any notable differences.

Blinding

Experimenters performed both surgical implantations and electrophysiological recordings, so it was not possible to be blinded to which face patch was implanted in each monkey.

Reporting for specific materials, systems and methods

We require information from authors about some types of materials, experimental systems and methods used in many studies. Here, indicate whether each material, system or method listed is relevant to your study. If you are not sure if a list item applies to your research, read the appropriate section before selecting a response.

Materials & experimental systems

n/a	Involved in the study	
x		Antibodies
x		Eukaryotic cell lines
x		Palaeontology and archaeology
	x	Animals and other organisms
x		Human research participants
x		Clinical data
x		Dual use research of concern

Methods

n/a	Involved in the study	
x		ChIP-seq
x		Flow cytometry
x		MRI-based neuroimaging

Animals and other organisms

Policy information about studies involving animals; ARRIVE guidelines recommended for reporting animal research

Laboratory animals	Four rhesus macaques (WA (male, 5 years old, 8.5 kg), MA (male, 7 y.o., 9.4 kg), SP (female, 15-16 y.o., 8.5 kg), MO (male, 4 y.o., 11 kg))
Wild animals	This study did not involve wild animals.
Field-collected samples	This study did not involve samples collected from the field.
Ethics oversight	All animal procedures and animal welfare were in full compliance with the Guidelines for the Care and Use of Laboratory Animals by National Institute of Health and approved by the Animal Care and Use Committee of the National Institute of Mental Health.

Note that full information on the approval of the study protocol must also be provided in the manuscript.
